# BRIDGE JOBS AS PATHS TO WORKING LONGER: DO GENDER AND RETIREMENT IDENTITIES MATTER?

**DOI:** 10.1093/geroni/igad104.0877

**Published:** 2023-12-21

**Authors:** Yun taek Oh, Phyllis Moen

**Affiliations:** University of Michigan, Ann Arbor, Michigan, United States; University of Minnesota, Minneapolis, Minnesota, United States

## Abstract

Leaving a full-time career job happens not only by completely exiting the labor force but also by having bridge jobs. When choosing to have bridge jobs, some workers perceive themselves as partially or completely retired while others are not. This study first categorizes bridge jobs into three types: switching occupations, leaving employers, and reducing work hours to part-time. Then, this study uses the Health and Retirement Study to investigate how the self-perceived retirement status in bridge jobs affects the timing of complete withdrawal from the labor force by using two-way fixed-effects event study regression. The results show that a significant proportion of older workers consider themselves partially or completely retired when leaving employers or reducing work hours but do not when switching occupations. The results also show the effects of bridge jobs on the complete withdrawal are different by the self-perceived retirement status and gender. Switching occupations reduces the probability of retirement for male workers who do not consider it as retirement while it increases the probability of retirement for female workers who consider it as retirement. Leaving the employer reduces the probability of retirement for male workers who consider these bride jobs as retirement while it does not for female workers. These findings imply the importance of self-perception of retirement in labor force participation and how it differs by the types of bridge jobs.

